# A High-Fidelity Phantom for the Simulation and Quantitative Evaluation of Transurethral Resection of the Prostate

**DOI:** 10.1007/s10439-019-02361-7

**Published:** 2019-09-18

**Authors:** Eunjin Choi, Fabian Adams, Stefano Palagi, Anina Gengenbacher, Daniel Schlager, Philippe-Fabian Müller, Christian Gratzke, Arkadiusz Miernik, Peer Fischer, Tian Qiu

**Affiliations:** 1 Micro Nano and Molecular Systems Lab, Max Planck Institute for Intelligent Systems, Heisenbergstr. 3, 70569 Stuttgart, Germany; 2 Department of Urology, University Medical Center Freiburg, Hugstetterstr. 55, 79106 Freiburg, Germany; 3 Institute for Physical Chemistry, University of Stuttgart, Pfaffenwaldring 55, 70569 Stuttgart, Germany

**Keywords:** Prostate, Organ phantom, Surgical simulation, Surgical evaluation, Endourology, Ultrasound imaging, 3D printing

## Abstract

Transurethral resection of the prostate (TURP) is a minimally invasive endoscopic procedure that requires experience and skill of the surgeon. To permit surgical training under realistic conditions we report a novel phantom of the human prostate that can be resected with TURP. The phantom mirrors the anatomy and haptic properties of the gland and permits quantitative evaluation of important surgical performance indicators. Mixtures of soft materials are engineered to mimic the physical properties of the human tissue, including the mechanical strength, the electrical and thermal conductivity, and the appearance under an endoscope. Electrocautery resection of the phantom closely resembles the procedure on human tissue. Ultrasound contrast agent was applied to the central zone, which was not detectable by the surgeon during the surgery but showed high contrast when imaged after the surgery, to serve as a label for the quantitative evaluation of the surgery. Quantitative criteria for performance assessment are established and evaluated by automated image analysis. We present the workflow of a surgical simulation on a prostate phantom followed by quantitative evaluation of the surgical performance. Surgery on the phantom is useful for medical training, and enables the development and testing of endoscopic and minimally invasive surgical instruments.

## Introduction

The prostate is an important gland in the male reproductive system. 90% of men by the age of 90 suffer from the benign enlargement of the prostate (benign prostate hyperplasia, BPH).^4^ BPH can be treated by prostatectomy and is often conducted in a minimally invasive way through the urethra. Procedures involve transurethral resection of the prostate (TURP) and holmium laser enucleation of the prostate (HoLEP),^19^ of which TURP has been the benchmark therapy for BPH.^3^,^17^ TURP involves the resection of the abnormally enlarged prostate tissue and involves the use of a wire loop that is heated by an alternating electrical current.^2^ Typically, a rigid endoscope is inserted through the urethra of the patient and the prostate tissue is resected under endoscopic observation. Two anatomical zones of the prostate are important for BPH: the peripheral zone and the central zone. Only the latter should be removed and the peripheral zone must be preserved. The two zones are, however, not easily distinguishable, as their appearance under endoscopic imaging is very similar. An experienced surgeon distinguishes the two zones based on the tactile feedback from the endoscope, as the tissues in the peripheral zone have higher elasticity.^14^ Thus, TURP calls for experienced surgical skills and intensive surgical training, especially as the surgery carries risks and 8–12% of the TURP patients need a follow-up surgical procedure.^18^

To lower the risk of surgical complications, it is promising to first perform surgical simulations, training and medical instrument testing on a phantom. Different phantom models have been developed and reported in the literature for many organs, such as blood vessel,^9^ kidney,^1^,^22^ brain,^15^,^24^ prostate,^5^,^7^,^12^,^16^*etc*. For example, Weinstock *et al.* built a brain phantom with realistic features for minimally invasive neurosurgery, using state-of-the-art 3D printing technology and special effects borrowed from the film industry.^24^ Recently, we developed a high-fidelity kidney phantom for endoscopy and ultrasound imaging training purposes using material molding technique.^1^ Betrouni *et al.* reported a prostate phantom for laser based thermotherapy treatment planning and simulation of the prostate cancer.^5^ For imaging applications, prostate phantoms are also fabricated^7^,^12^ and commercially available (Limbs & Things Inc., Savannah, GA, USA & CIRS, Norfolk, VA, USA), but they either neglect mechanical properties or are made of materials (e.g. polyvinyl chloride, polymethacrylate) that are not suitable for electrocautery, as they release toxic gases when heated. Although phantoms have been designed for surgical simulation purposes, they normally require video review and the evaluation of the surgical performance only involves subjective scoring.^6^,^10^ Existing methods for quantitatively measuring TURP performance are limited and not particularly precise as they are restricted to post-surgery determination of resected tissue weight^21^ and surgical time.^13^ To our knowledge, there is no existing method for quantitative evaluation of the overall tissue resection performance in an objective manner, and there is no phantom model of the prostate that permits such an evaluation. With the fast development of minimally invasive surgical technologies, especially the rise of surgical robotics, there is a real need for quantitative surgical evaluation methods, both for medical device testing and surgical training. Realistic organ models also enable the development of new medical instruments. Here, we present such a phantom.

Our phantom was fabricated by a two-step molding process using 3D printed molds. The phantom is made from non-toxic biomimetic hydrogels, which are engineered to match the mechanical property of normal and BPH tissues. Ultrasound contrast agents, that are not detectable in endoscopy, are added to distinguish the peripheral zone and the central zone for post-operative quantitative evaluation.

Three operators with different levels of surgical experience performed the TURP respectively on identical phantoms and their surgical performance could be visualized and evaluated using ultrasound imaging. 3D reconstructed ultrasound images were then analyzed to objectively and quantitatively evaluate the surgical performance. We show that it is now straightforward to define precise evaluation criteria. The quantitative evaluation of the surgical performance presents a unique way to assess surgical procedures and the surgical skills of surgeons, which is not possible with a real patient.

## Materials and Methods

### Design of the 3D Digital Model of the Prostate

**Figure 1:**
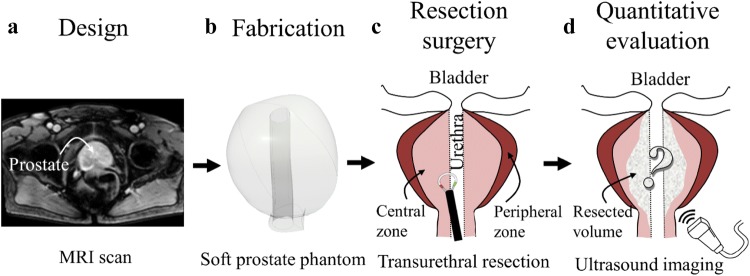
General scheme of a prostate phantom for surgical simulation and evaluation, which consists of the following steps: (a) The phantom is designed based on data of magnetic resonance imaging (MRI); (b) The phantom is fabricated using 3D printing and molding method with soft tissue-like materials; (c) Surgery process, such as transurethral resection of prostate surgery (TURP), is performed on the phantom; (d) The surgical outcome is quantitatively evaluated by an imaging method, such as ultrasound imaging.

**Figure 2:**
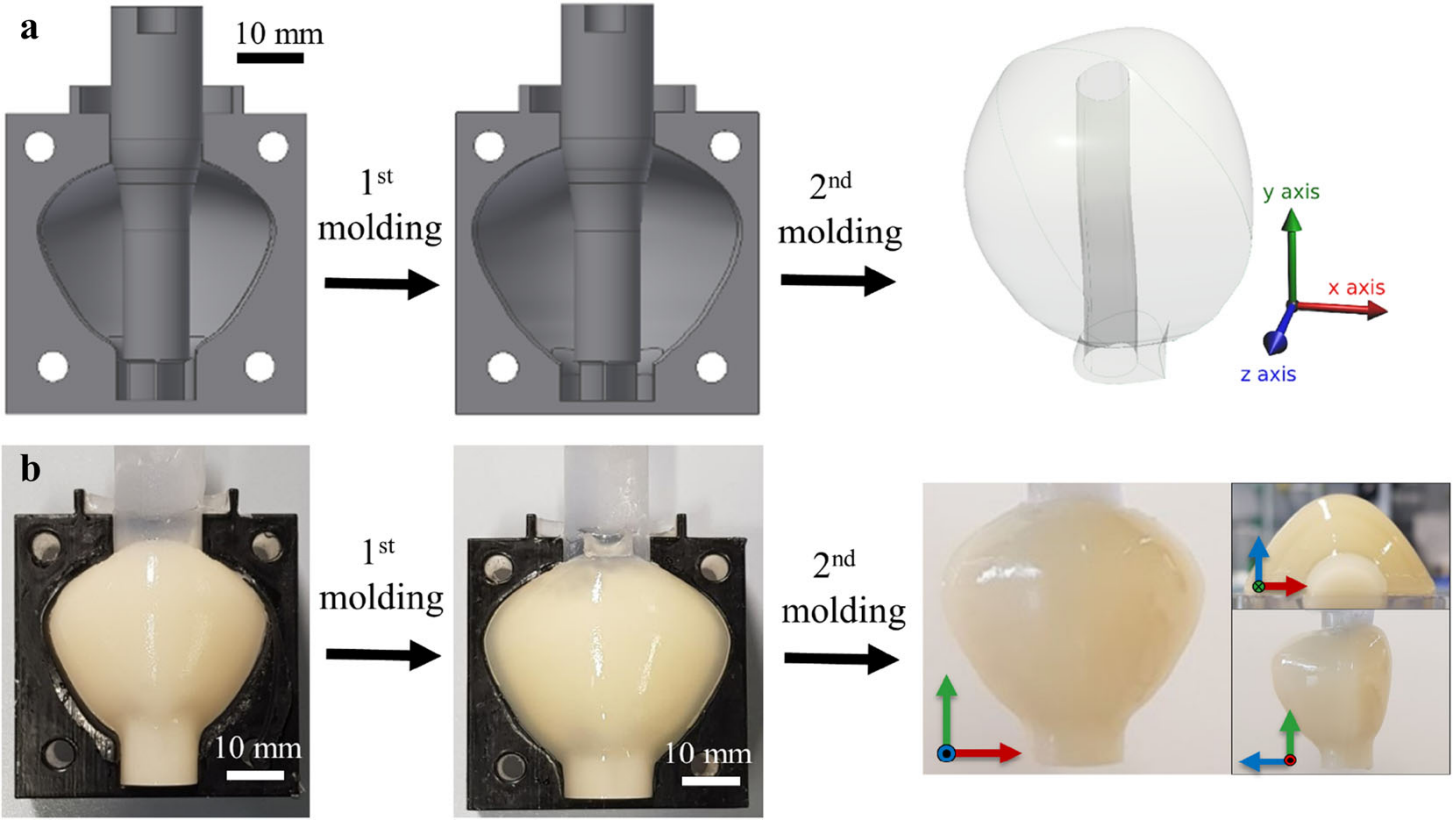
Fabrication process of the prostate phantom. (a) Schematic showing the 2-step molding process with two 3D-printed molds to fabricate peripheral zone and central zone; (b) Photographic illustration of the molding process and the resulting prostate phantom is shown along three orthogonal directions.

MRI (Magnetic Resonance Imaging) images of the prostate were obtained from an online database (Prostate MR Image Database, http://prostatemrimagedatabase.com/, accessed on April 10, 2016) and served as models for the design of a generalized digital model of the prostate (Fig. 1). As the prostate’s size and shape differ in patients, an average volume of ~ 20 cm^3^ (this corresponds to a bounding box of 40 mm × 32 mm × 35 mm in W × L × H) was used to design the three-dimensional (3D) digital model. Important anatomical structures, i.e., the peripheral zone, the central zone and the urethra, were designed using the computer aided design (CAD) software (Inventor 2016, Autodesk Inc., San Rafael, CA, USA). Complementary molds were designed for the molding of the central and the peripheral zone, respectively (Fig. 2a). Each mold consists of three parts: a lower and upper half separated by the bisecting central plane and an insert at the center that represents the urethra.

### Fabrication of the Prostate Phantom

The molds for the prostate phantom were printed using a 3D printer (Object 260 Connex, Stratasys, Israel). VeroClear material (Stratasys, Israel) was used to for the main part of the mold, as the molding process can be observed through the transparent material. TangoBlackPlus material (Stratasys, Israel) was used on the surface of the lower mold as a sealing layer between the two-half molds assembly (Fig. 2b). To allow the use of biomimetic materials, direct 3D printing must be complemented with molding, as we have previously shown that commercial 3D printing materials cannot reproduce all the properties required for surgery of human tissue.^1^ We filled the molds with materials that possess the desired visual, haptic, and imaging properties. The fabrication process is shown in Fig. 2. The central zone was first molded in the first mold with a central plug forming the urethra. After curing, it was detached from the first outer mold and molded in a second larger mold to form the peripheral zone with a second material.

An aqueous solution of 4% (w/v) poly(vinyl alcohol) (PVA, MW = 89,000 − 98,000, > 99% hydrolyzed, Sigma-Aldrich, Germany) was prepared at 90 °C under stirring overnight as a stock solution. The prepared PVA stock solution was heated and diluted to 1.25% (w/v). Then, agar powder (fine powder, FCC, Sigma-Aldrich, Germany) was added to prepare 0.75, 1.00, 1.25, 1.50, 1.75% (w/v) mixture solutions. The solution was heated in a microwave oven to completely dissolve the agar powder. Subsequently, 1.5% (w/v) hollow glass powder (~ 20 μm in diameter, iM16K, 3M™ glass bubbles, 3M, Maplewood, MN, USA), which is a strong ultrasound contrast material, was added to the solution for the central zone, but not the peripheral zone. The mixture was filled in the first mold for the inner layer with the cylindrical insert for the urethra and kept at 4 °C for 0.5 h for curing. The outer layer was fabricated using the second mold by covering the fabricated inner layer in the same procedure using a mixture with a different agar concentration and without the ultrasound contrast agent. To fix the phantom for surgical simulation and ultrasound imaging, the phantom was embedded in 2.5% (w/v) agar. The fabricated phantom was then placed in a rectangular box (50 mm × 50 mm × 95 mm in W × L × H) and the agar solution was poured into the box to completely immerse the phantom. Then, the box was placed into an ice bath until the solution solidified. The model was stored at 4 °C. All solutions were prepared with aqueous solution of 0.9% (w/v) NaCl (≥ 99.5%, Roth, Germany) to provide similar electrical conductivity as that of human tissue. The fabricated layers were easily taken out from the molds and the insert was finally removed by gently pulling. In total fifteen phantoms were fabricated.

### Mechanical Testing

**Figure 3:**
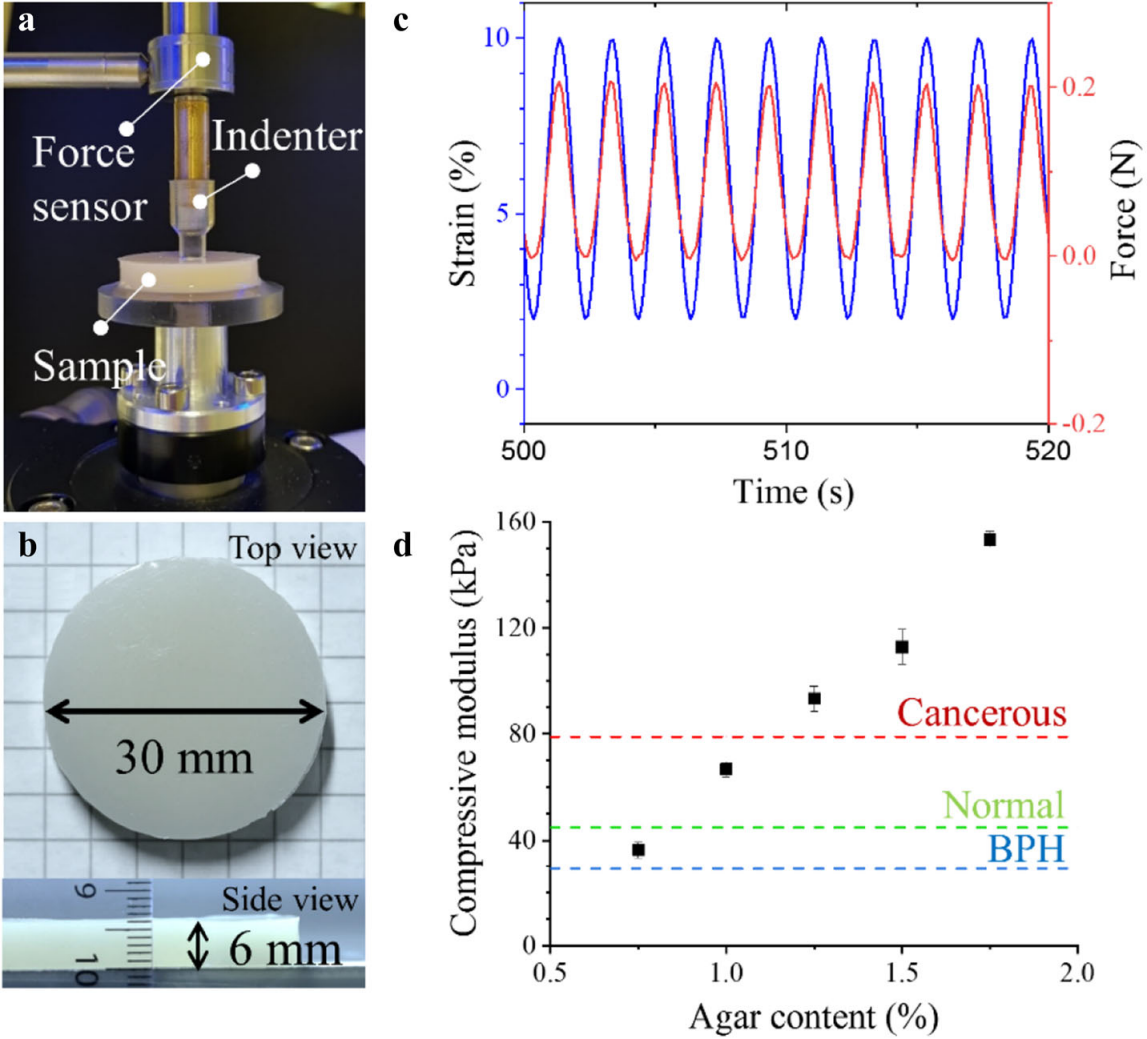
Mechanical property of the phantom materials. (a) The experimental setup for measuring the compressive elastic modulus using an indentation method; (b) Size and shape of the test sample used for measurements; (c) The strain and force under cyclic load are in phase; (d) Measured compressive elastic modulus (*E*) of the phantom materials using the indentation method and comparison with the range of human prostate tissues: average values for cancerous, normal and benign prostate hyperplasia (BPH) tissues are indicated.

The mechanical property of the mixture was tested by a mechanical testing machine (ElectroForce 3200 series test instrument, TA instruments, New Castle, DE, USA). As shown in Fig. 3a, a customized cylindrical indenter with a radius of 3 mm was fabricated with the VeroClear material by 3D printing (Object 260 Connex, Stratasys), and connected to a force sensor (maximum detectable force of 22.2 N, ElectroForce Systems Group, TA instruments, New Castle, DE, USA). Samples of phantom materials were fabricated in cylindrical poly(methyl methacrylate) (PMMA) molds of 6 mm in thickness and 15 mm in radius and applied in between the holder and the indenter (Figs. 3a and 3b). Cyclic strain of 3, 5, 8% was, respectively, applied at 0.5 Hz under 2% pre-compression. The temperature during the measurements was kept at 23.0 ± 0.1 °C. Ten full cycles were applied as the initial step, and the data was taken at the steady state from twenty full cycles. The compressive elastic modulus by indentation was calculated using the Eq. (1),^14^ where a Poisson’s ratio of 0.495 is assumed for an incompressible material^8^:

1 $$E = \frac{{2\left( {1 - \nu ^{2} } \right)qa}}{w}$$

where *v* is Poisson’s ratio, *q* is load density, *a* is the loaded area, and *w* is the maximum displacement.

### Ultrasound Imaging

**Figure 4:**
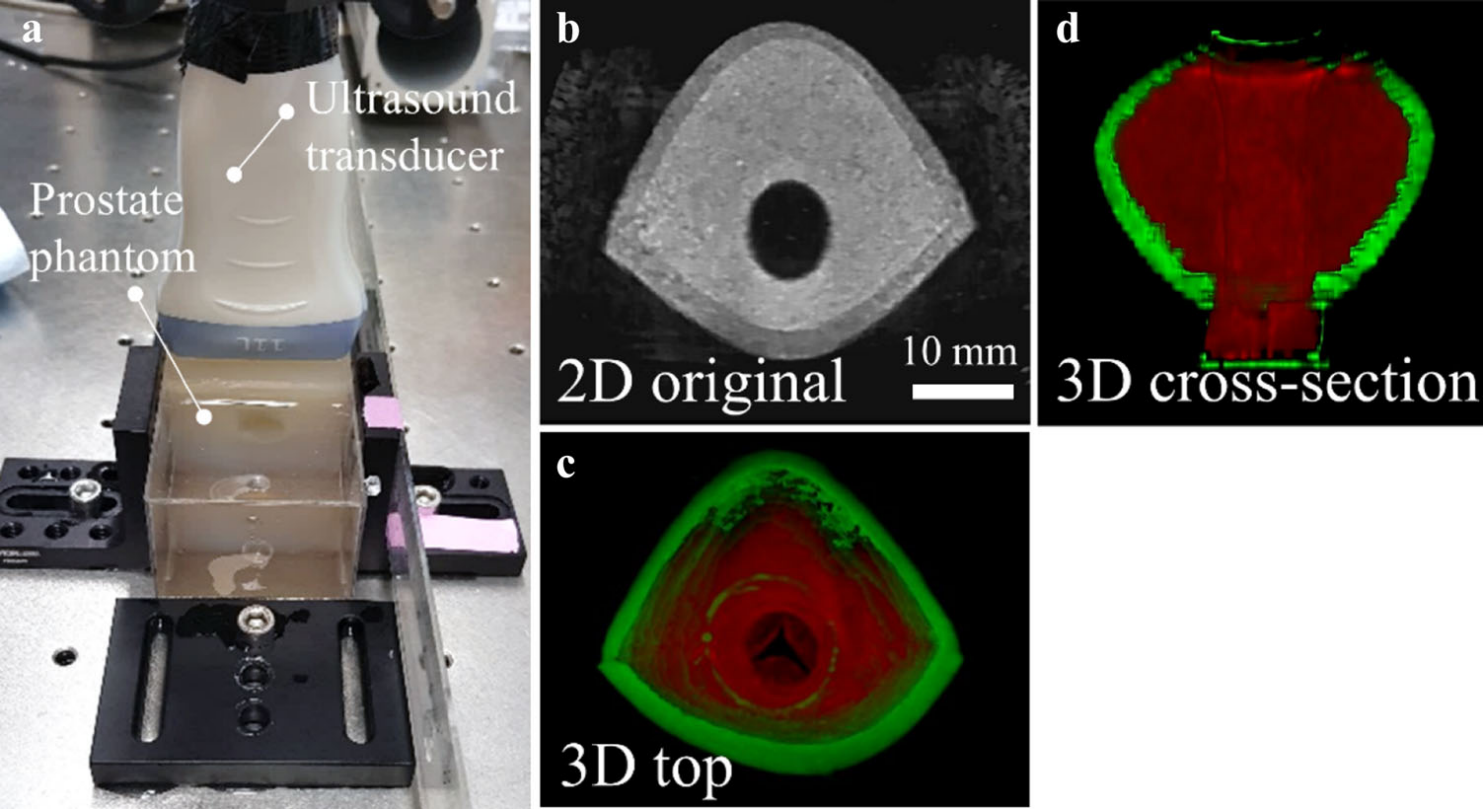
Validation of the prostate phantom by ultrasound imaging. (a) The set-up for automatic scanning stage for ultrasound imaging of the phantom in the front view; (b) The cross-sectional ultrasound image of prostate phantom in two dimension (2D); (c) and (d) Reconstructed 3D model of the prostate phantom in pseudo color from the top view (c) and the cross-sectional view (d). The red and green colors represent the central zone and the peripheral zone, respectively.

The phantoms were imaged with a clinical ultrasound imaging device (LOGIO P6, GE Healthcare, Chicago, IL, USA) and a linear array ultrasound transducer (L11, 10 MHz, GE Healthcare Japan Corporation, Japan) before and after the surgery. The phantom was placed under water and fixed on a linear stage (Thorlabs, Newton, NJ, USA) to facilitate imaging (Fig. 4). Multiple linear scans along the direction of the urethra were automatically acquired in 0.5 mm intervals (3D sound field scanner, GAMPT mbH, Germany). Image processing was used to increase the image contrast between the central and peripheral zones (Photoshop CS5.1, Adobe Inc., San Jose, CA, USA). For automatic detection, image segmentation, area extraction and calculation were processed by a customized code in Matlab (R2018a, MathWorks, Natick, MA, USA), and 3D image reconstruction were conducted using Fiji (ImageJ 1.51p, NIH, Bethesda, MD, USA).

### TURP on the Prostate Phantom

**Figure 5:**
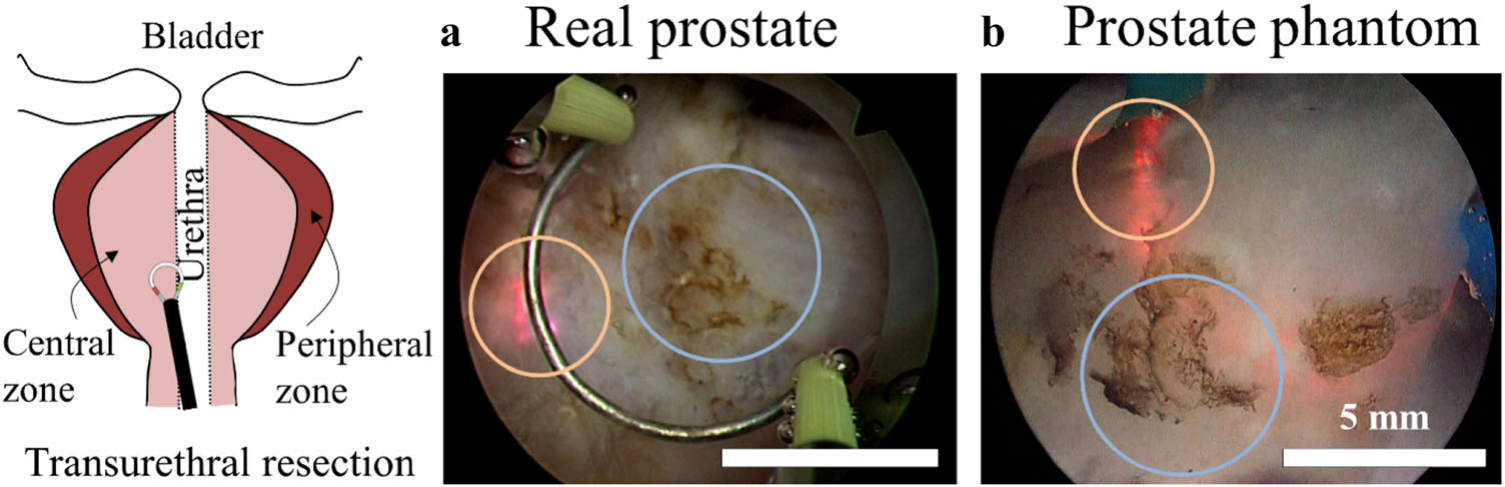
Optical resemblance of resection behavior on transurethral resection under endoscope. The endoscopic view of the transurethral resection of prostate surgery (TURP) on a real human prostate (a, a snapshot from a YouTube video) and the prostate phantom (b). The orange and blue circles label the electrical spark around the electrode and the scar after electrocautery, respectively.

**Figure 6:**
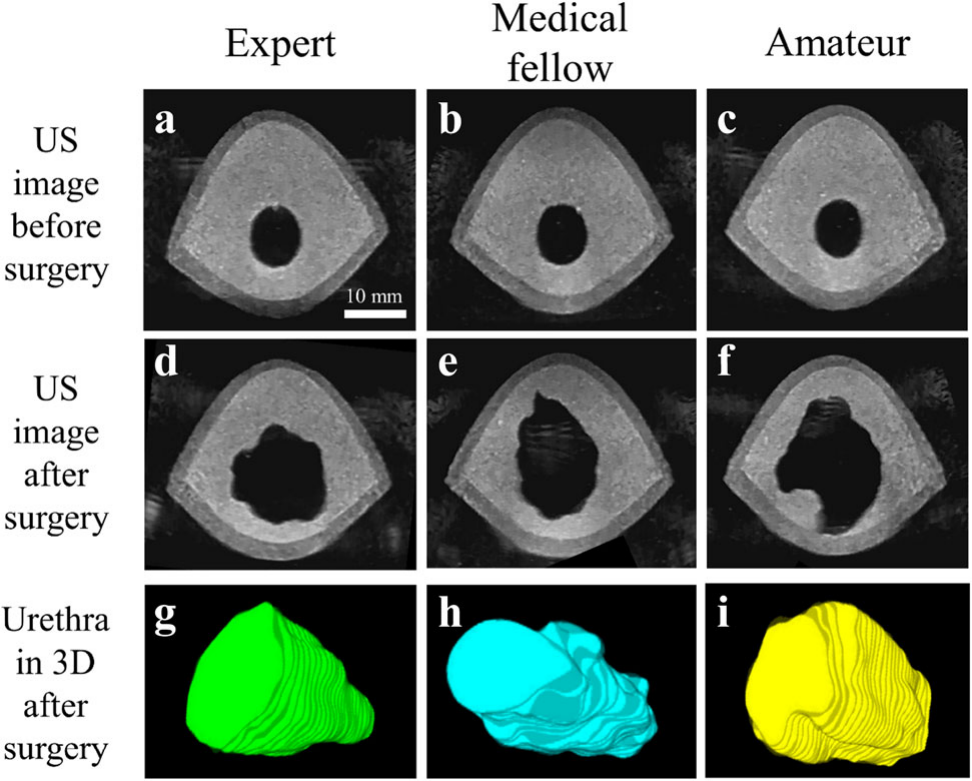
Comparison of ultrasound (US) images before and after surgical simulation resulting from different skilled participants, who are classified as an expert (a, d, g), a medical fellow (b, e, h) and an amateur (c, f, i). The different prostate phantom are almost identical which is confirmed using US images (a–c). After surgery, US images (d–f) and 3D reconstructed images of the resected volume along the urethra (g–i) show large difference in the surgical performance of the participants.

TURP was performed using a standard 26 Fr. transurethral bipolar resectoscope (120 W, Karl Storz, Germany). TURP was conducted on nine identical prostate phantoms, by three different skilled participants: a surgeon (expert), a medical fellow, and an untrained operator (amateur). The surgeon, medical fellow and the amateur each performed TURP on three prostate phantoms, respectively (Figs. 5 and 6).

### Evaluation Parameters

**Figure 7:**
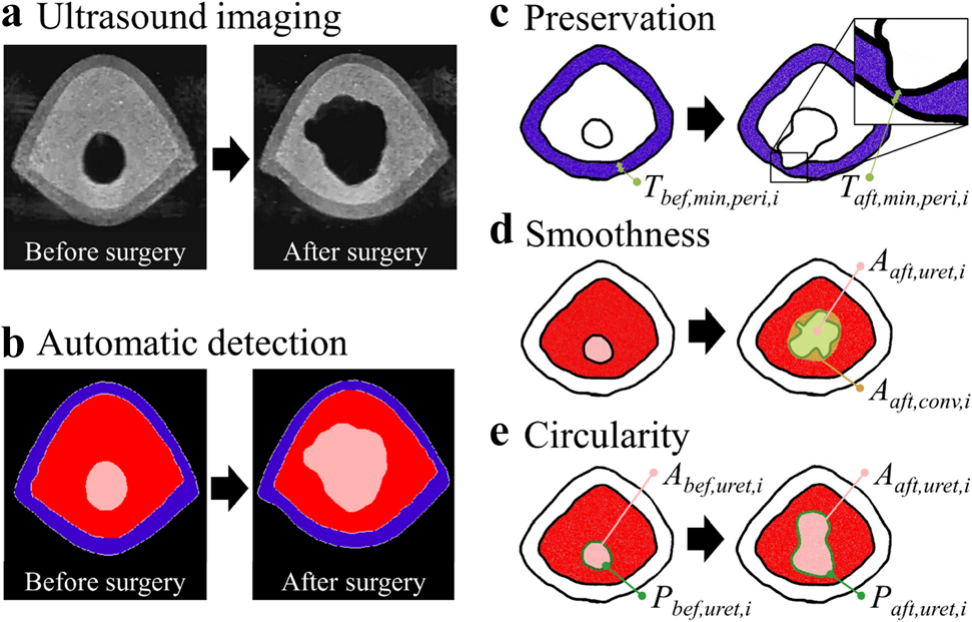
Evaluation process and parameters for the surgical simulation; (a) The phantoms are imaged using ultrasound scanning before and after surgery; (b) The interesting regions are segmented by automated detection (blue: peripheral zone, red: central zone and pink: urethra zone) and corresponding values are extracted to evaluate surgical skills based on three parameters (c–e); (c) The preservation of peripheral zone; (d) The smoothness of resection boundary; (e) The circularity of resection.

Quantitative parameters of the surgical performance can be evaluated by analyzing the ultrasonic images. Three parameters: (a) preservation of the peripheral zone, (b) smoothness of the resection boundary, and (c) circularity of the resection area, were determined in consultation with surgeons. They are defined as is shown in Fig. 7.

The first criterion is that the peripheral zone (outer layer of the phantom) is fully preserved during TURP. Since the thickness of the outer layer varies along the urethra, the reference point is the minimum thickness of the layer in each imaging slice. The minimum thicknesses before ($T_{\text{bef, min, peri,i}}$) and after ($T_{\text{aft,min,peri,i}}$) surgery in the *i*-th imaging slice were obtained using automatic detection from ultrasonic images, respectively. The overall preservation ratio of the peripheral zone ($\bar{R}_{\text{Pres}}$) was calculated and averaged for *n* = 153 slices by the Eq. (2).

2 $$\bar{R}_{Pres} = \frac{1}{n}\mathop \sum \limits_{i = 1}^{n} \frac{{T_{aft,min,peri, i} }}{{T_{bef,minperi, i} }}$$

As a second parameter, the smoothness of the resection boundary was characterized by the ratio given by the resection area of the urethra (*A*_aft,ureth,i_) to its convex area (*A*_aft_,_conv,i_) after surgery from each slice of prostate phantoms.^20^ The average ratio of the smoothness ($\bar{R}_{\text{Smoo}}$) of all *i*-th imaging slices indicates smoothness of resection trajectory and was calculated using Eq. (3).

3 $$\bar{R}_{\text{Smoo}} = \frac{1}{n}\mathop \sum \limits_{i = 1}^{n} \frac{{A_{\text{aft,uret,i}} }}{{A_{\text{aft,conv,i}} }}$$

The third parameter that was defined and evaluated is the circularity of the resected area. TURP surgery aims at enlarging the urethra space which is blocked by the BPH tissue. The circularity parameter describes how symmetrically the resection is performed along the urethra.

4 $$C_{\text{bef,i}} = \frac{{4\pi A_{\text{bef,uret,i}} }}{{P_{\text{bef,uret,i}}^{2} }}$$

The circularity before surgery (*C*_bef,i_) was calculated using the resection area of the urethra (*A*_bef,uret,i_) and the perimeter of the resection area (*P*_bef,uret,i_) using the Eq. (4)^20^ along the *i*-th imaging slice. The circularity after surgery (*C*_aft,i_) was processed in same manner. Then, the average circularity ($\bar{R}_{\text{Circ}}$) was calculated using the Eq. (5).

5 $$\bar{R}_{\text{Circ}} = \frac{1}{n}\mathop \sum \limits_{i = 1}^{n} \frac{{C_{\text{aft,i}} }}{{C_{\text{bef,i}} }}$$

**Figure 8:**
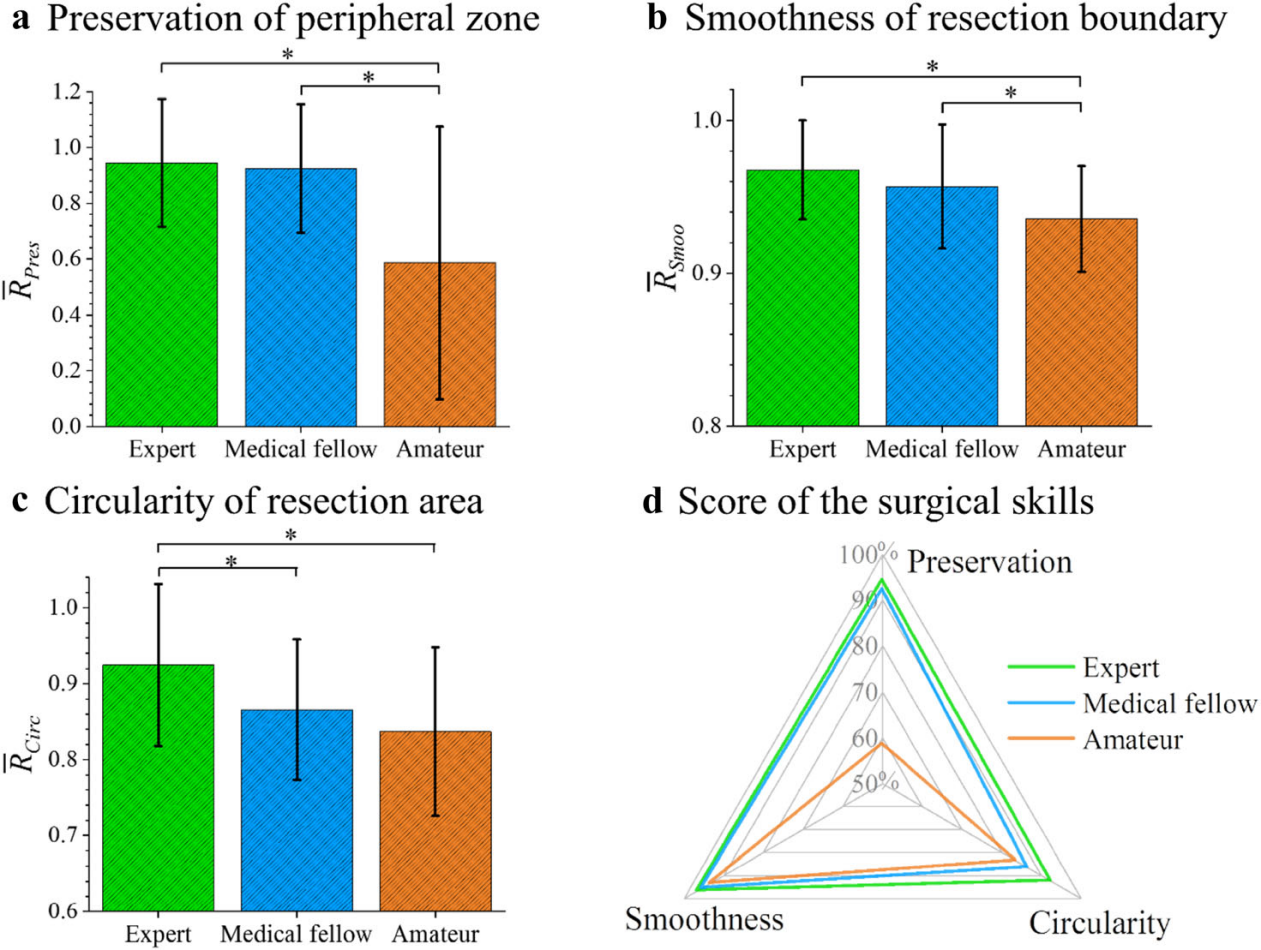
Quantitative evaluation of the surgical skills; (a) Preservation of the peripheral zone ($\bar{R}_{pres}$) is a safety parameter related to the safety of the surgery; (b) Smoothness of resection boundary ($\bar{R}_{smoo}$) describes a dexterous control of the surgical tools, which results in a smooth resection boundary; (c) Circularity of resection area $\left( {\bar{R}_{circ} } \right)$ is about the symmetry of the resection around the urethra. The error bars represent standard deviations and the values are compared using *t* test (*p < 0.01); (d) The evaluated surgical skills based on the three parameters are displayed as a scoring system in a radar chart.

Statistical analysis was performed using two-sample two-tailed *t* tests with a *p* value of 0.01 for significant difference (Excel, Microsoft, USA). The averaged values of the three parameters are normalized and quoted as a percentage (100%). The radar chart of Fig. 8 allows for a comparison of these criteria and an evaluation of the different surgeries as well as the overall surgical skill of the three participants.

## Results

### Design and Fabrication of the Prostate Phantom

To simulate TURP, the prostate phantom consists of important anatomical structures, including the urethra and the two important zones of the prostate, i.e. the peripheral zone and the central zone. To give a realistic haptic response, the elasticity of the peripheral zone and the central zone are tuned using different concentration of materials and are therefore molded separately (see “Materials and Methods” section, and Fig. 2a). While exhibiting different elasticities, they are nevertheless indistinguishable in their appearance under endoscopic view, as required. Our methods permit the fabrication of high-fidelity organ phantoms to the designed shape. As shown in the pictures of the fabricated phantom (Fig. 2b, right), the central zone is fully covered by the peripheral zone, and the shape of both layers can be fully replicated.

### Validation of the Phantom Materials

A TURP instrument uses electric current to generate heat for the electrocautery of tissue, and it is thus important that the materials for the phantom match the electrical conductivity and thermal conductivity of real human prostate tissue. This could be reproduced by preparing the hydrogel (agar) in a NaCl electrolyte solution. In addition, the surgery relies on haptic information which requires mechanical differences in the tissue. Since pure agar gel is brittle and does not offer the toughness under resection, PVA was added to the agar.^23^ The amount of agar is important for the overall mechanical property of the material.^11^

The elastic modulus of the phantom materials were compared with reported moduli of prostate tissues^6^ and measured *in vitro* using the same measuring method. To measure the elastic modulus of the phantom materials, only a small strain was applied to avoid the viscous influence of the viscoelastic phantom materials, such that no phase shift between the strain and force (stress) curves was observed under cyclic loading (Fig. 3c). The determined elasticity of the phantom materials: 1.25% (w/v) of PVA and with 0.75, 1.00, 1.25, 1.50, and 1.75% (w/v) of agar, is plotted in Fig. 3d and shows that increased agar content leads to higher compression moduli, leading to the stages from BPH, to normal, and to cancerous prostate tissues.^14^ Thus, the materials containing 0.75 and 1.25% of agar were selected for the central and the peripheral zone, respectively. Finally, microscale hollow glass particles are added only to the material of the central zone. They are too small to be detected in the endoscopic view by the surgeon (Fig. 5), but they provide a clear contrast during ultrasound imaging, which benefits the evaluation.

### Validation of the Phantom Anatomy by Ultrasound Imaging

The fabricated phantom was embedded in agar and framed to scan by clinical ultrasound machine (Fig. 4a). The central zone containing the ultrasound contrast agent is clearly brighter than the peripheral zone without the glass particles. The urethra appears black as the phantom is immersed in water (Fig. 4b). The central zone is shown in red (false color) and the green area represents the peripheral zone as seen in Figs. 4c and 4d. Both are in good agreement with the designed shape of the phantom.

### Surgical Simulation on the Phantom

The fabricated phantom shows very similar tissue resection to TURP on human tissue (orange circle in Fig. 5a, a snapshot from an online video at https://www.youtube.com/watch?v=8JrHdcqpn_0, accessed on Nov. 6, 2017). The cut phantom tissue also shows scarring as in a corresponding real prostate surgery (Fig. 5, blue circle). The scars serve as visual aids to help surgeons with the orientation of the instrument, and to make the simulated surgical scene more realistic.

The 2D ultrasound images at the same location of the phantom before and after surgery and consequent 3D reconstructed images of the resected section along the urethra are shown in Fig. 6. Comparison of the three phantoms before surgery shows that they are almost identical, suggesting the high reproducibility of the fabrication process. This feature of our fabrication process is important, as it permits comparative and quantitative surgical evaluations. Qualitative differences can clearly be observed. The TURP by the expert and the medical fellow is circular. In contrast, the surgery by the amateur was not successful as it shows a dangerous cut too close to the peripheral zone. Besides the qualitative observation of the images, the organ phantom developed here offers much more insight into the surgical procedure, which is absent in traditional surgical training.

### Quantitative Evaluation of Surgical Performance

To quantify the surgical performance of the prostate phantom, we introduce automated image detection and quantitative evaluation parameters. Segmented image areas: urethra (labeled pink in image), central zone (labeled red) and peripheral zone (labeled blue) were extracted automatically from the ultrasound images (Figs. 7a and 7b). The automatically detected areas were then evaluated using three parameters: preservation of the peripheral zone, smoothness of the resection boundary and circularity of the resection area.

The preservation of the peripheral zone is defined by Eq. (2), as the average ratio of the minimum thicknesses of the peripheral zone before and after surgery (*T*_bef,min,peri,i_ and *T*_aft,min,peri,i_) (Fig. 7c). Considering the perfect value of the ratio is 1.0 when the peripheral zone experiences no damage, one sees that the expert and the medical fellow both achieved a high ratio over 0.9, which corresponds to a successful intervention (Fig. 8a). On the other hand, the amateur penetrated the peripheral zone at multiple points and the ratio of 0.5 shows larger variation along the urethra. The second parameter we define evaluates the smoothness of the resection boundary, which is calculated as the ratio of the resected area over its convex area (Fig. 7d). The ideal value of the parameter is 1, i.e., the resected boundary is in a completely smooth convex shape. The results in Fig. 8b show that the amateur performed worst with the lowest value indicating relatively poor control of the instrument during TURP. The third parameter is the circularity of the resection area as defined in Eq. (4) (Fig. 7e). As seen in Fig. 8c, the circularity of the resection area decreases with the expertise in surgery from expert to amateur with significant difference. A detailed analysis along the urethra reveals that the resection by the medical fellow is smooth, but not quite circular. The cut of the amateur is irregular with a rough boundary and, importantly, shows zones damage to the peripheral zone which can lead to complications.

Each quantitative parameter only reflects one aspect of the surgery. We define a normalized score for each criterion using percentage: 100% as a maximum. The score matrices are presented in a radar chart in Fig. 8d. This chart gives feedback to the user on different aspects of their surgical performance, and provides some useful insight in which particular direction the surgical skills should be improved. The amateur clearly shows the lowest scores and hence performance in all aspects, and the medical fellow and the surgeon show similar performance over two parameters, but differ in the circularity of the resection area.

## Discussion

Each evaluation parameter is carefully selected with experienced surgeons, and they indicate the important aspects of the TURP surgical skills. The preservation of the peripheral zone is the most important parameter, as it corresponds to safety of the surgery. A safety margin should be preserved at all times in the TURP. Although neither of the medical fellow or the amateur has experience with TURP, the medical fellow had knowledge of the anatomy and a basic understanding of the surgical procedure, thus the medical fellow achieved a high score regarding the safety parameter. The second parameter of the resection boundary smoothness relates to the precise control of the medical instrument during the surgery. Since surgeons indirectly contact the tissues with minimally invasive tools and have a restricted vision, a smooth resection requires experience and sophisticated handling of the instrument to provide equal forces in all directions during resection. The third parameter of the circularity of the resected area presents an advanced criterion for the surgery. The resected area should be all around the blocked urethra, and opens up the urethra area in a circular symmetrical manner. It was the only parameter among the three that can distinguish the performance between the expert and the medical fellow with a significant difference, which suggests that following the right direction for resection along the urethra is a difficult task and requires extensive practice. The surgical simulation could help surgeons and medical students to familiarize themselves with the instrument and to improve their surgical skills before operating on patients. These evaluation parameters only serve as examples to show that the performance of the surgery can be analyzed in an automatic and subjective manner in a phantom like the one we demonstrate here.

The two-step fabrication method of 3D printing and molding reported herein is general, so that customized organ models with different shapes and material properties can be produced with similar approaches. By changing the concentration of the hydrogel, its elasticity can be tuned over a large range, thus mimicking a variety of tissue conditions, including normal, BPH and cancerous prostate tissue. The variations in different zones as well as individual patients can be taken into account for a personalized phantom. Future study will compare real human organs with the phantom to further improve its fidelity.

In summary, we report a novel approach to fabricate high-fidelity organ phantom for the quantitative evaluation of the surgical performance. The prostate phantom is made of inexpensive and biomimetic hydrogel materials that mimic the important physical properties (mechanical, haptic, visual, and behavior under electrocautery) of human tissue for the simulation of transurethral resection of the prostate (TURP). Using contrast agents that are selectively visible and give high contrast under ultrasonic imaging, values of specific regions are extracted using automated image analysis in both pre and post-operative examinations. A major advance is the use of imaging contrast agents that are not visible to the surgeon during surgery, but that can be used for post-surgical evaluation. The surgeries can therefore be evaluated using quantifiable performance criteria. This offers a unique way to train, plan, and evaluate surgical procedures that cannot be monitored with real patients. The phantom can be used in routine clinical practice in the near future. Other quantitative parameters can also be defined and be used to evaluate, train and improve the surgical skills according to specific surgical procedures. Surgery on identical and realistic phantoms will thus not only be useful for surgical training, but also facilitate the testing and development of new medical instruments, including surgical robots.
